# Feminizing and masculinizing gender-affirming hormone therapy affects fibrin clot characteristics in opposite directions

**DOI:** 10.1016/j.rpth.2026.106648

**Published:** 2026-05-08

**Authors:** Mette Bøgehave, Dorte Glintborg, Louise Lehmann Christensen, Guy T'Sjoen, Martin den Heijer, Marianne Skovsager Andersen, Else-Marie Bladbjerg

**Affiliations:** 1 Department of Clinical Biochemistry, Unit for Thrombosis Research, University Hospital of Southern Denmark, Esbjerg, Denmark; 2 Department of Regional Health Research, University of Southern Denmark, Odense, Denmark; 3 Department of Endocrinology, Odense University Hospital, Odense, Denmark; 4 Department of Clinical Research, University of Southern Denmark, Odense, Denmark; 5 Department of Endocrinology and Center for Sexology and Gender, Ghent University Hospital, Ghent, Belgium; 6 Department of Endocrinology and Metabolism & Center of Expertise on Gender Dysphoria, Amsterdam UMC, Amsterdam, the Netherlands

**Keywords:** estradiol, fibrin, testosterone, thrombosis, transgender persons

## Abstract

**Background:**

The mechanisms behind the increased occurrence of thrombosis associated with gender-affirming hormone treatment (GAHT) are not clarified. We hypothesized that GAHT alters fibrin clot characteristics (formation, lysis, and structure) in a prothrombotic direction.

**Objectives:**

To investigate changes in *ex vivo* fibrin clot characteristics after 12 months of feminizing or masculinizing GAHT.

**Methods:**

We included 251 transgender women and 320 transgender men aged >17 years in a prospective cohort study. Clot formation (velocity [*V*_max_], maximum absorbance [MA], and overall hemostasis potential [OHP]), clot lysis, and structure (fiber density and diameter) were studied by turbidity at baseline and after 12 months of feminizing GAHT (3 groups of oral/transdermal estradiol and cyproterone acetate) or masculinizing GAHT (7 groups of intramuscular/transdermal testosterone).

**Results:**

In transgender women, feminizing GAHT increased *V*_max_, MA, and OHP, while clot lysis decreased (total group, all *P* < .001). Oral estradiol was associated with the largest changes (Δ0-12 months: ΔOHP, 6.03 optical density × min; *P* = .02; Δclot lysis: −5.0%; *P* = .005). Fiber density and diameter remained unchanged. In transgender men, *V*_max_, MA, OHP, and fiber diameter decreased following masculinizing GAHT, whereas clot lysis and fiber density increased (total group, all *P* < .001). We observed no between-group differences in clot characteristics at 12 months or for changes (Δ0-12 months).

**Conclusion:**

Feminizing GAHT for 12 months had prothrombotic effects on the fibrin clot, whereas masculinizing GAHT had antithrombotic effects. The procoagulant effect was most evident with oral feminizing GAHT. This large cohort study provides important insights into the mechanisms linking GAHT to thrombotic risk.

## Essentials

• GAHT may alter thrombotic risk, but effects on fibrin clot properties are unclear.
• Fibrin clot formation, structure, and lysis were assessed before and after 12 months of GAHT.
• Feminizing GAHT increased fibrin formation and reduced clot lysis.
• Masculinizing GAHT reduced fibrin formation and enhanced clot lysis.

## 1 Introduction

Gender-affirming hormone therapy (GAHT) is a cornerstone in transgender care, enabling individuals to align their physical appearance with their gender identity [1]. Transgender women receive feminizing GAHT with estradiol and antiandrogens, while transgender men receive masculinizing GAHT with testosterone [1]. It is of increasing importance to understand the safety of GAHT because a growing number of individuals are treated with GAHT worldwide [2,3].

Both transgender women and transgender men have an increased occurrence of venous and arterial thrombosis compared with cisgender men and women, respectively [[4], [5], [6], [7]]. GAHT is believed to contribute to this elevated risk, although the underlying mechanisms remain incompletely understood [6,7]. We have recently demonstrated that the thrombin generation is increased after 12 months of feminizing GAHT but reduced after masculinizing GAHT [8]. Thrombin is responsible for fibrin formation, but it is unknown how GAHT affects the formation, lysis, and structure of fibrin, a major component of a thrombus.

The turbidimetric clot formation and lysis assay provides a dynamic assessment of fibrin clot properties, including fibrin formation, structure of the fibrin clot, and susceptibility to clot lysis. Prothrombotic clot profiles are characterized by increased maximum absorbance (MA), maximal turbidity increment (*V*_max_), overall hemostasis potential (OHP; increased fibrin formation), increased fiber diameter, reduced fiber mass density (altered protein packing within individual fibers), and resistance to fibrinolysis (reduced clot lysis) [[9], [10], [11], [12], [13]]. Fibrinogen levels influence these measures [14,15] and may be altered by GAHT. Indeed, fibrinogen increases following feminizing GAHT [16,17], whereas no consistent changes have been observed following masculinizing GAHT [18]. However, it remains unclear how GAHT affects the structural and functional properties of the fibrin network.

Data on fibrin clot characteristics in transgender individuals are limited. One small study found no difference in OHP between transgender women treated with estradiol (and in 14 of 26 combined with cyproterone acetate [CPA]) and cisgender men, but increased fibrin clot lysis in transgender women [19]. A study in cisgender individuals has shown that the use of ethinylestradiol and progestin in oral contraceptives increased MA, fiber diameter, and fiber mass density [20], whereas use of anabolic androgenic steroids, including testosterone, reduced clot lysis without affecting *V*_max_, fiber diameter, or fiber mass density [21]. These inconsistent findings indicate a need for larger prospective studies with well-defined GAHT regimens to clarify how feminizing and masculinizing GAHT influence fibrin clot characteristics.

The overall aim of this prospective 1-year cohort study was to evaluate how GAHT in different administration forms affect fibrin clot characteristics. We hypothesize that GAHT is associated with prothrombotic effects in transgender women and transgender men, based on the assumed GAHT-induced increased thrombotic risk in transgender women and transgender men.

## 2 Methods

### 2.1 Participants and study design

The present study is part of the European Network for the Investigation of Gender Incongruence, a multicenter prospective cohort study among collaborating gender identity clinics. The European Network for the Investigation of Gender Incongruence study includes individuals with a confirmed gender dysphoria/incongruence diagnosis, assessed eligible for treatment, without prior GAHT use, and with proficient knowledge of the native language. All participants provided informed consent [22,23].

The participants and design of the present study are described in detail elsewhere [8]. In brief, we included 618 participants aged >17 years who visited the clinics in Amsterdam and Ghent between April 2012 and March 2020 with blood samples collected prior to and 12 months after GAHT therapy. We excluded participants receiving estrogen-containing oral contraceptives or any form of antiandrogen therapy other than CPA. None of the participants received anticoagulant or antifibrinolytic medication or underwent gender-affirming genital surgery during follow-up. The final cohort comprised 270 transgender women (Amsterdam: *n* = 182; Ghent: *n* = 88) and 348 transgender men (Amsterdam: *n* = 223; Ghent: *n* = 125). Comorbidities and use of medication at study inclusion are presented in Supplementary Table 1.

### 2.2 Ethics

The Ethical Committee of Ghent University Hospital, Belgium, approved the overall study protocol with additional approvals obtained from the local ethical committees of each participating clinical center.

### 2.3 Treatment protocol

The treatment protocol is described in detail elsewhere [8,23]. Briefly, GAHT was administrated according to the standards of care version 7 of the World Professional Association for Transgender Health [24]. Feminizing GAHT consisted of the antiandrogen CPA (10-50 mg/d) in combination with an estradiol agent, targeting serum concentrations of estradiol and testosterone within the reference interval for adult cisgender women. Estradiol was administrated either as oral estradiol valerate at a daily dose of 2 to 6 mg, with 2 mg twice daily as the most commonly prescribed regimen (E oral + CPA), or as transdermal estradiol patches of 50 to 150 μg/24 h (E transdermal + CPA). Some participants shifted between oral and transdermal estradiol during follow-up, starting with transdermal estradiol followed by oral administration, or vice versa (E oral/transdermal + CPA).

Masculinizing GAHT consisted of testosterone, administrated intramuscularly as testosterone undecanoate 1000 mg every 10 to 12 weeks, with a second injection given after 6 weeks (T intramuscular [i.m.] undecanoate), as i.m. testosterone ester 125 to 250 mg every 2 to 3 weeks (T i.m. ester), or as transdermal testosterone gel 25 to 75 mg daily (T transdermal). Additionally, 4 subgroups received various types of testosterone, alternating among undecanoate, ester, and gel preparations. One subgroup did not receive synthetic gestagen (progestin, G; T mixed), 1 subgroup initiated progestin at some point during 12 months (T mixed + G), 1 subgroup received progestin at baseline only (G_0 + T mixed), and 1 subgroup received progestin at baseline and at some point during 12 months (G_0 + T mixed + G). Progestin (administrated orally, intrauterine, or by injection) was prescribed to transgender men for pregnancy prevention, menstrual suppression, or management of breakthrough bleeding [1].

### 2.4 Characteristics of participants

Table 1 presents baseline characteristics of participants for the entire study cohort (E total in transgender women and T total in transgender men) and stratified by GAHT modality. Participant characteristics before and 12 months after GAHT have previously been published [8,25,26] and are presented in Supplementary Tables 2 and 3. In brief, in transgender women, body mass index, sex hormone–binding globulin (SHBG), and 17β-estradiol levels increased, whereas hematocrit, testosterone, total cholesterol, low-density lipoprotein (LDL) cholesterol, and triglyceride levels decreased in the E total group. Effects differed between GAHT modalities for SHBG, 17β-estradiol, and cholesterols [8]. In transgender men, body mass index, hematocrit, testosterone, total cholesterol, LDL cholesterol, and triglyceride levels increased, while SHBG, 17β-estradiol, and high-density lipoprotein cholesterol levels decreased in the T total group. Effects differed between GAHT modalities for SHBG, 17β-estradiol, and cholesterols [8].

**Table 1:** Baseline characteristics and gender-affirming hormone therapy in transgender women and transgender men.

Transgender	*N* (Ams/Ghe)	Age (y)	Smokers (%)	Gender affirming hormone therapy	Group name
Women	270 (182/88)	25 (21, 35)	20	Total estradiol group	E total
	175 (100/75)	23 (20, 28)	20	Estradiol valerate 2-6 mg/d and CPA 10-50 mg/d	E oral + CPA
	77 (68/9)	38 (27, 44)^a^	22	Estradiol patches 50-150 μg/d and CPA 10-50 mg/d	E transdermal + CPA
	18 (14/4)	27 (22, 43)	11	Estradiol valerate/patches^b^ and CPA 10-50 mg/d	E oral/transdermal + CPA
Men	348 (223/125)	21 (19, 26)	24	Total testosterone group	T total
	85 (27/58)	22 (19, 26)	24	Testosterone i.m. undecanoate 1000 mg/10-12 wk	T i.m. undecanoate
	50 (50/0)	21 (19, 25)	24	Testosterone i.m. ester 125-250 mg/2-3 wk	T i.m. ester
	45 (45/0)	22 (20, 27)	22	Testosterone gel 25-75 mg/d	T transdermal
	39 (38/1)	21 (20, 26)	28	Testosterone undecanoate/ester/gel^b^	T mixed
	37 (20/17)	21 (19, 28)	27	Testosterone undecanoate/ester/gel^b^ and progestin	T mixed + G
	70 (24/46)	21 (19, 24)	26	Baseline progestin + testosterone undecanoate/ester/gel^b^	G_0 + T mixed
	22 (19/3)	22 (19, 24)	14	Baseline progestin + testosterone undecanoate/ester/gel^b^ + progestin	G_0 + T mixed + G

Age is presented as median (25, 75 percentile) and was compared between hormone groups using a Kruskal–Wallis test. Smoking is presented as current smokers in percentage of the total group and was compared between hormone groups using a chi-squared test. Previously published by Bøgehave et al. [8].

Ams, Amsterdam; CPA, cyproterone acetate; E, estradiol; G, injected, intrauterine, or oral progestin; G_0, progestin at baseline; Ghe, Ghent; i.m., intramuscular; T, testosterone.

a Age is highest in the E transdermal + CPA group of transgender women (Mann–Whitney test, *P* < .05).

b Shifting over time between oral and transdermal therapy (in transgender women) or between testosterone undecanoate and ester and gel (in transgender men).

### 2.5 Blood sampling

Venous blood samples were collected from the antecubital vein at baseline and 12 months after GAHT, following a period of rest in a seated position. Samples were drawn into tubes containing 0.105 M/0.109 M sodium citrate. Platelet-poor plasma was prepared by centrifugation at 1800 × *g* for 10 minutes. Plasma was rapidly stored at −80 °C in tightly capped cryotubes until analysis. Samples were thawed in a water bath at 37 °C and analyzed within 1 hour in 1 series for each participant.

### 2.6 Plasma analyses

We determined *ex vivo* plasma fibrin polymerization and clot lysability by turbidity measurements as described by Sjøland et al. [14] with a few adjustments. In brief, fibrin polymerization was assessed by mixing citrated plasma with thrombin (1.0 IU/mL, final concentration) and CaCl_2_ (15 mmol/L, final concentration). Fibrin clot lysis was investigated simultaneously in a different well by adding tissue plasminogen activator (300 ng/mL, final concentration). Turbidity was measured as optical density (OD) at 340 nm every 15 seconds for 30 minutes on a Sunrise plate reader (Tecan Group Ltd). A clot-lysis curve was created (Supplementary Figure) from which the following variables were obtained: the steepest slope of the OD curve recorded during polymerization (*V*_max_, OD/min); MA (OD), calculated as the difference between the highest point on the lysis curve and absorbance value at time = 0; OHP (OD × minutes), calculated as the area under the clot-lysis curve and reflecting the balance between fibrin generation and lysis; and fibrin clot lysis (in percentage), reflecting the percentage of fibrin lysed in 30 minutes. To determine fibrin clot structure, the plate with the fibrin clot was sealed and incubated overnight at 25 °C to obtain a stable clot. OD was read at 340, 405, 540, 608, and 690 nm and fibrin fiber diameter (in micrometers) and fiber mass density (×10^22^ Da/cm^3^) were calculated according to the studies by Carr and Hermans [27] and Carr and Gabriel [28]. Interassay coefficients of variation for the fibrin clot characteristics were 12% for *V*_max_, 3% for MA, 7% for OHP, 15% for clot lysis, and 7% for fiber diameter and fiber mass density.

Plasma levels of fibrinogen (in grams per liter) were determined with fibrinogen polyclonal antibodies (Siemens Healthcare Diagnostics Products GmbH) using the Behring Nephelometer II analyzer (Siemens Healthcare Diagnostics Products GmbH). Precision of the assay was 3% for interassay variation. Plasma analyses were performed in 251 transgender women (Amsterdam: *n* = 165; Ghent: *n* = 86) and 320 transgender men (Amsterdam: *n* = 197; Ghent: *n* = 123), reflecting the availability of citrated plasma.

### 2.7 Statistical analysis

Plasma fibrin clot characteristics were compared between baseline and 12 months (within-group changes) with a paired *t*-test or Wilcoxon signed-rank test. Between-group comparisons (between GAHT administration forms) at baseline were performed with an analysis of variance for normally distributed variables and a Kruskal–Wallis test for skewed variables. Between-group comparisons at 12 months were performed using a linear regression analysis, adjusted for baseline values and in transgender women also for confounding effects of between-group differences in age. Between-group comparisons for absolute changes (Δ, 0-12 months) and relative changes (Δ0-12 months/0 months) were performed using linear regression analysis. When the assumptions of linear regression were not met, either a log-transformation of the dependent variable or robust SEs were applied to account for violations of normality and/or heteroscedasticity. When significant between-group effects were observed in the analysis of variance or in the linear regression analysis, pairwise comparisons were performed with the Bonferroni test.

Results are presented as mean (95% CI) or median (25, 75 percentiles) as appropriate. A *P* value of <.05 was considered statistically significant. All statistical analyses were performed using Stata 18 (StataCorp LLC).

The present analysis is an efficacy analysis with the aim to determine treatment effects of GAHT, and data were analyzed according to a per-protocol analysis on study completers. An intention-to-treat analysis was not feasible, as plasma fibrin clot characteristics were only measured in participants who adhered to their assigned GAHT throughout follow-up.

### 3 Results

#### 3.1 Transgender women

Measures of fibrin clot characteristics at baseline and 12 months after GAHT are presented in Table 2. From baseline to 12 months, we observed a significant increase in *V*_max_, MA, OHP, and fibrinogen in the combined cohort (E total) and in all GAHT subgroups, except that increases in OHP and fibrinogen were not significant in the transdermal group. We observed a significant decrease in clot lysis in E total and E oral + CPA subgroups. Fibrin fiber diameter and fiber mass density did not change significantly. We observed no significant between-group differences at 12 months. Between-group differences in absolute changes (Δ0-12 months) were observed for OHP (*P* = .02), fibrinogen (*P* = .001), and clot lysis (*P* = .007; Table 2), driven by a larger increase (ΔOHP: 6.03 OD × min; 95% CI, 0.80-11.25; *P* = .02; Δfibrinogen: 0.3 g/L; 95% CI, 0.1-0.5; *P* = .001) and a larger decrease (Δclot lysis: −5.0%; 95% CI, −8.8 to −1.2; *P* =.005) in the E oral + CPA group than in the E transdermal + CPA group. Between-group differences in relative changes (Δ0-12 months/0 months) were observed for MA (*P* = .03), OHP (*P* = .001), clot lysis (*P* = .008), and fibrinogen (*P* < 0.001), with a trend toward significance for *V*_max_ (*P* = .06; Figure). This was due to a larger increase (ΔMA: 8.7%; 95% CI, 0.7-16.7; *P* = .03; ΔOHP: 15.0%; 95% CI, 5.1-25.0; *P* = .001; Δfibrinogen: 12.0%; 95% CI, 5.3-18.7; *P* < .001; Δ*V*_max_: 9.2%; 95% CI, −0.2 to 18.6; *P* = .06), and a larger decrease (Δclot lysis: −7.2%; 95% CI, −12.7 to −1.7; *P* = .006) in the E oral + CPA group compared with that in the E transdermal + CPA group.

**Table 2:** Fibrin clot characteristics in transgender women before and 12 months after gender-affirming hormone therapy.

Variable	0 mo	12 mo	*P*	Change (0-12 mo)
*V*_max_ (OD/min)^a^^,^^b^				
E total (*n* = 251)	0.67 (0.65 to 0.69)	0.78 (0.75 to 0.80)	**<.001**	0.11 (0.08 to 0.13)
E oral + CPA (*n* = 163)	0.64 (0.62 to 0.67)	0.77 (0.73 to 0.80)	**<.001**	0.13 (0.10 to 0.15)
E transdermal + CPA (*n* = 71)	0.74 (0.70 to 0.79)^c^	0.81 (0.76 to 0.85)	**.02**	0.06 (0.01 to 0.11)
E oral/transdermal + CPA (*n* = 17)	0.63 (0.56 to 0.70)	0.71 (0.65 to 0.78)	**.02**	0.09 (0.02 to 0.15)
MA (OD)^b^				
E total (*n* = 250)	0.56 (0.54 to 0.57)	0.63 (0.61 to 0.65)	**<.001**	0.07 (0.06 to 0.09)
E oral + CPA (*n* = 163)	0.53 (0.51 to 0.55)	0.62 (0.60 to 0.64)	**<.001**	0.09 (0.07 to 0.10)
E transdermal + CPA (*n* = 71)	0.62 (0.59 to 0.66)^c^	0.66 (0.64 to 0.69)	**.02**	0.04 (0.01 to 0.08)
E oral/transdermal + CPA (*n* = 16)	0.53 (0.48 to 0.58)	0.61 (0.56 to 0.66)	**.01**	0.08 (0.02 to 0.13)
OHP (OD × min)^a^^,^^b^				
E total (*n* = 251)	48.66 (46.72 to 50.61)	56.45 (54.56 to 58.34)	**<.001**	7.79 (6.03 to 9.54)
E oral + CPA (*n* = 163)	45.68 (43.51 to 47.85)	55.25 (52.89 to 57.61)	**<.001**	9.57 (7.56 to 11.58)
E transdermal + CPA (*n* = 71)	56.28 (52.13 to 60.44)^c^	59.83 (56.20 to 63.47)	.07	3.54 (−0.21 to 7.29)^d^
E oral/transdermal + CPA (*n* = 17)	45.43 (39.44 to 51.43)	53.85 (47.53 to 60.16)	**.006**	8.41 (3.16 to 13.67)
Clot lysis (%)				
E total (*n* = 251)	74.6 (72.6 to 76.6)	71.4 (69.5 to 73.3)	**<.001**	−3.2 (−4.6 to −1.8)
E oral + CPA (*n* = 163)	76.5 (74.2 to 78.8)	71.8 (69.5 to 74.1)	**<.001**	−4.7 (−6.5 to −2.9)
E transdermal + CPA (*n* = 71)	70.3 (66.4 to 74.3)^d^	70.6 (67.0 to 74.4)	.81	0.3 (−2.1 to 2.7)^d^
E oral/transdermal + CPA (*n* = 17)	74.0 (64.6 to 83.4)	71.0 (62.7 to 79.3)	.21	−3.0 (−7.4 to 1.5)
Fiber diameter (μm)^a^^,^^b^				
E total (*n* = 227)	0.13 (0.13 to 0.13)	0.13 (0.13 to 0.14)	.05	0.00 (−0.00 to 0.01)
E oral + CPA (*n* = 149)	0.13 (0.13 to 0.13)	0.13 (0.13 to 0.14)	.13	0.00 (−0.00 to 0.01)
E transdermal + CPA (*n* = 63)	0.13 (0.12 to 0.13)	0.13 (0.13 to 0.13)	.53	0.00 (−0.00 to 0.01)
E oral/transdermal + CPA (*n* = 15)	0.13 (0.12 to 0.14)	0.14 (0.12 to 0.16)	.21	0.01 (−0.01 to 0.03)
Fiber density (× 10^22^ Da/cm^3^)^a^^,^^b^^,^^e^^,^^f^				
E total (*n* = 227)	4.6 (4.1 to 5.2)	4.6 (4.2 to 5.1)	.57	0.0 (−0.5 to 0.4)
E oral + CPA (*n* = 149)	4.6 (4.2 to 5.3)	4.6 (4.2 to 5.1)	.33	0.0 (−0.6 to 0.4)
E transdermal + CPA (*n* = 63)	4.6 (4.2 to 5.0)	4.7 (4.3 to 5.1)	.29	0.1 (−0.3 to 0.6)
E oral/transdermal + CPA (*n* = 15)	4.9 (3.9 to 5.2)	4.2 (3.8 to 4.9)	.24	−0.2 (−0.7 to 0.2)
Fibrinogen (g/L)^a^^,^^b^				
E total (*n* = 249)	2.6 (2.6 to 2.7)	2.9 (2.8-2.9)	**<.001**	0.2 (0.2 to 0.3)
E oral + CPA (*n* = 163)	2.5 (2.4 to 2.6)	2.8 (2.7-2.9)	**<.001**	0.3 (0.3 to 0.4)
E transdermal + CPA (*n* = 69)	2.9 (2.8 to 3.1)^g^	3.0 (2.8-3.1)	.75	0.0 (−0.1 to 0.2)^d^
E oral/transdermal + CPA (*n* = 17)	2.6 (2.4 to 2.8)	2.9 (2.7-3.1)	**.02**	0.2 (0.1 to 0.4)

Values presented as mean (95% CI) were compared at 0 and 12 months (within-group changes) with a paired *t*-test. Changes (0-12 months) are presented as mean (95% CI). Between-group comparisons (between gender-affirming hormone treatment administration forms) at 0 months were performed with an analysis of variance. Between-group comparisons at 12 months were performed with a linear regression analysis, adjusted for baseline values (0 months) and age. Changes (0-12 months) were compared between gender-affirming hormone treatment administration forms using multivariate linear regression analysis. When significant between-group effects were observed in the analysis of variance or linear regression analysis, pairwise comparisons were performed with the Bonferroni test. Oral/transdermal refers to oral and transdermal estradiol shifting over time. *P* values shown in bold are statistically significant (*P* <.05).

CPA, cyproterone acetate; E, estradiol; MA, maximum absorbance; OD, optical density; OHP, overall hemostasis potential.

a Between-group comparisons at 12 months were performed with a multivariate linear regression analysis with log-transformed outcome variables or robust SEs to meet model assumptions.

b Changes (0-12 months) were compared between gender-affirming hormone treatment administration forms using multivariate linear regression analysis with robust SEs to meet model assumptions.

c Significantly higher than E oral + CPA and E oral/transdermal + CPA.

d Significantly lower than E oral + CPA.

e Values presented as median (25, 75 percentiles) were compared at 0 and 12 months (within-group changes) with a Wilcoxon signed-rank test and between-group comparisons at 0 months were performed with a Kruskal–Wallis test.

f Changes (0-12 months) are presented as median (25, 75 percentiles).

g Significantly higher than E oral + CPA.

**Figure:**
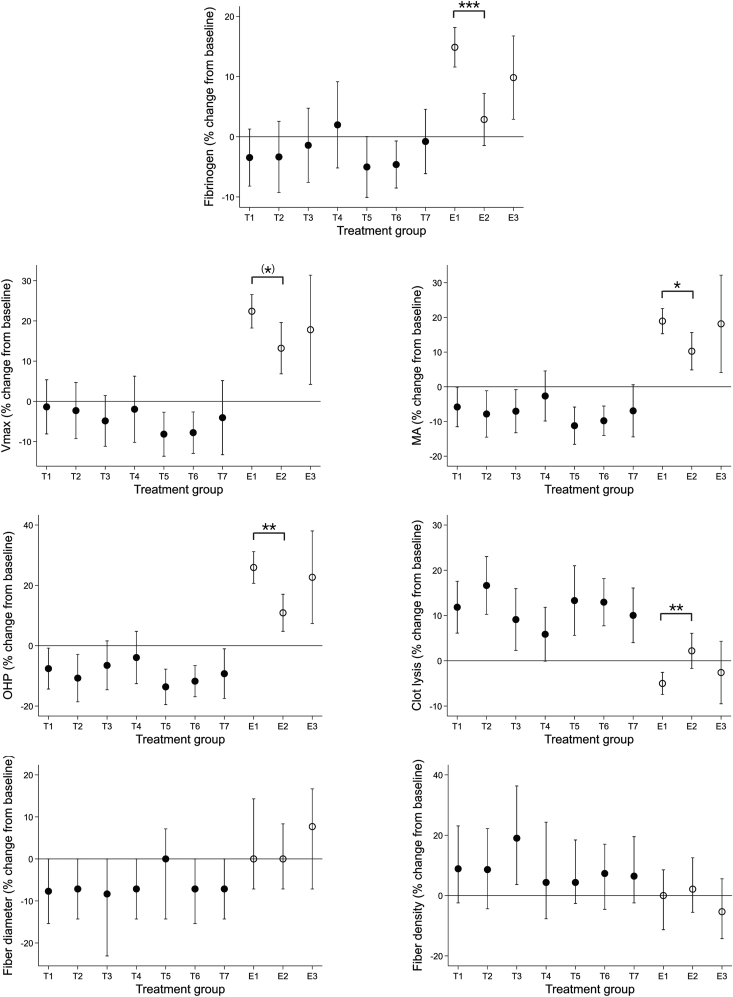
Relative changes in measures of fibrin clot characteristics from baseline to 12 months. Results are presented as mean with 95% CI or median with 25, 75 percentiles (fiber diameter and density). (∗)*P* = .06; ∗*P* < .05; ∗∗*P* < .01; ∗∗∗*P* < .001. T1: T intramuscular undecanoate; T2: T intramuscular ester; T3: T transdermal; T4: T mixed; T5: T mixed + G; T6: G_0 + T mixed; T7: G_0 + T mixed + G; E1: E oral + cyproterone acetate (CPA); E2: E transdermal + CPA; E3: E oral/transdermal + CPA (group abbreviations are defined in Table 1). MA, maximum absorbance; OHP, overall hemostasis potential; ○, feminizing gender-affirming hormone treatment; •, masculinizing gender-affirming hormone treatment.

To explore whether the observed changes in fibrin clot properties were driven by fibrinogen levels, turbidity variables were normalized by calculating the ratio between each clot variable and the corresponding fibrinogen concentration. Fiber diameter and fiber mass density were not normalized to fibrinogen concentration, as fibrinogen is incorporated into the calculation of these structural measures. After this normalization, the longitudinal changes over 12 months were generally comparable with the unadjusted results, with the direction of effects remaining consistent despite minor differences in statistical significance. For between-group comparisons of absolute changes, the previously observed difference in OHP between oral and transdermal estradiol was no longer significant, whereas MA became significantly different between groups (*P* = .04). Fibrinogen-normalized results are provided in Supplementary Table 4.

### 3.2 Transgender men

Measures of fibrin clot characteristics at baseline and at 12 months after GAHT are presented in Table 3. From baseline to 12 months, we observed a significant decrease in *V*_max_, MA, OHP, fiber diameter, and fibrinogen, as well as a significant increase in clot lysis and fiber mass density in the combined cohort (T total) and across subgroups, although not all changes reached statistical significance within the subgroups.

**Table 3:** Fibrin clot characteristics in transgender men before and 12 months after gender-affirming hormone therapy.

Variable	0 mo	12 mo	*P*	Change (0-12 mo)
*V*_max_ (OD/min)				
T total (*n* = 320)	0.78 (0.75 to 0.80)	0.72 (0.70 to 0.74)	**<.001**	−0.06 (−0.08 to −0.04)
T i.m. undecanoate (*n* = 83)	0.74 (0.70 to 0.79)	0.69 (0.65 to 0.73)	.07	−0.05 (−0.10 to 0.00)
T i.m. ester (*n* = 47)	0.81 (0.75 to 0.87)	0.77 (0.71 to 0.82)	.07	−0.04 (−0.09 to 0.00)
T transdermal (*n* = 40)	0.78 (0.71 to 0.85)	0.72 (0.66 to 0.78)	**.04**	−0.06 (−0.11 to −0.01)
T mixed (*n* = 32)	0.78 (0.69 to 0.86)	0.74 (0.67 to 0.80)	.19	−0.04 (−0.11 to 0.02)
T mixed + G (*n* = 33)	0.78 (0.70 to 0.85)	0.70 (0.64 to 0.76)	**.003**	−0.08 (−0.13 to −0.03)
G_0 + T mixed (*n* = 67)	0.77 (0.73 to 0.81)	0.69 (0.65 to 0.73)	**<.001**	−0.07 (−0.11 to −0.34)
G_0 + T mixed + G (*n* = 18)	0.87 (0.74 to 1.00)	0.81 (0.69 to 0.93)	.15	−0.06 (−0.14 to 0.19)
MA (OD)				
T total (*n* = 320)	0.66 (0.65 to 0.68)	0.60 (0.58 to 0.61)	**<.001**	−0.07 (−0.08 to −0.05)
T i.m. undecanoate (*n* = 83)	0.64 (0.61 to 0.67)	0.58 (0.55 to 0.61)	**.002**	−0.06 (−0.09 to −0.02)
T i.m. ester (*n* = 47)	0.69 (0.64 to 0.74)	0.62 (0.57 to 0.66)	**.001**	−0.07 (−0.11 to −0.03)
T transdermal (*n* = 40)	0.62 (0.57 to 0.67)	0.56 (0.52 to 0.59)	**.007**	−0.06 (−0.11 to −0.02)
T mixed (*n* = 32)	0.65 (0.60 to 0.70)	0.62 (0.58 to 0.66)	.16	−0.03 (−0.08 to 0.01)
T mixed + G (*n* = 33)	0.69 (0.64 to 0.75)	0.60 (0.56 to 0.64)	**<.001**	−0.09 (−0.14 to −0.05)
G_0 + T mixed (*n* = 67)	0.67 (0.63 to 0.70)	0.59 (0.56 to 0.62)	**<.001**	−0.07 (−0.10 to −0.05)
G_0 + T mixed + G (*n* = 18)	0.74 (0.66 to 0.81)	0.67 (0.61 to 0.73)	**.04**	−0.06 (−0.12 to −0.01)
OHP (OD × min)^a^				
T total (*n* = 320)	60.73 (58.83 to 62.63)	53.15 (51.51 to 54.79)	**<.001**	−7.58 (−9.23 to −5.92)
T i.m. undecanoate (*n* = 83)	58.18 (54.88 to 61.49)	51.29 (48.09 to 54.50)	**<.001**	−6.89 (−10.78 to −3.00)
T i.m. ester (*n* = 47)	64.08 (58.45 to 69.72)	55.04 (50.11 to 59.97)	**<.001**	−9.04 (−13.44 to −4.64)
T transdermal (*n* = 40)	55.62 (49.53 to 61.71)	49.38 (45.24 to 53.53)	**.01**	−6.23 (−10.76 to −1.70)
T mixed (*n* = 32)	59.39 (53.77 to 65.00)	54.94 (50.38 to 59.49)	.10	−4.45 (−9.64 to 0.74)
T mixed + G (*n* = 33)	64.71 (58.43 to 70.98)	54.50 (49.41 to 59.59)	**<.001**	−10.21 (−14.76 to −5.65)
G_0 + T mixed (*n* = 67)	61.25 (57.16 to 65.35)	52.87 (49.03 to 56.71)	**<.001**	−8.38 (−11.41 to −5.34)
G_0 + T mixed + G (*n* = 18)	68.16 (59.25 to 77.07)	60.52 (53.17 to 67.87)	**.02**	−7.64 (−13.47 to −1.81)
Clot lysis (%)				
T total (*n* = 320)	67.2 (65.3 to 69.1)	73.1 (71.4 to 74.8)	**<.001**	5.9 (4.7 to 7.1)
T i.m. undecanoate (*n* = 83)	68.7 (65.6 to 71.4)	74.5 (71.7 to 77.4)	**<.001**	5.9 (3.3 to 8.4)
T i.m. ester (*n* = 47)	63.6 (58.0 to 69.1)	71.9 (66.6 to 77.2)	**<.001**	8.4 (5.1 to 11.6)
T transdermal (*n* = 40)	72.3 (66.1 to 78.5)	76.1 (71.2 to 81.0)	**.03**	3.8 (0.4 to 7.2)
T mixed (*n* = 32)	68.5 (63.5 to 73.4)	71.5 (66.5 to 76.6)	.09	3.1 (−0.4 to 6.5)
T mixed + G (*n* = 33)	63.6 (57.8 to 69.4)	70.0 (64.7 to 75.2)	**.002**	6.3 (2.6 to 10.0)
G_0 + T mixed (*n* = 67)	66.4 (61.9 to 70.9)	73.1 (68.6 to 77.5)	**<.001**	6.6 (4.2 to 9.1)
G_0 + T mixed + G (*n* = 18)	66.1 (57.6 to 74.5)	71.3 (63.4 to 79.1)	**.006**	5.2 (1.9 to 8.6)
Fiber diameter (μm)^a^^,^^b^				
T total (*n* = 295)	0.14 (0.13 to 0.14)	0.13 (0.12 to 0.13)	**<.001**	−0.01 (−0.01 to −0.01)
T i.m. undecanoate (*n* = 74)	0.13 (0.13 to 0.14)	0.12 (0.12 to 0.13)	**.02**	−0.01 (−0.02 to −0.00)
T i.m. ester (*n* = 44)	0.14 (0.14:0.15)	0.14 (0.12:0.15)	.21	−0.01 (−0.02 to 0.00)
T transdermal (*n* = 36)	0.13 (0.12 to 0.14)	0.11 (0.10 to 0.12)	**<.001**	−0.02 (−0.03 to −0.01)
T mixed (*n* = 27)	0.14 (0.14 to −0.15)	0.13 (0.12 to 0.14)	**.01**	−0.01 (−0.02 to −0.00)
T mixed + G (*n* = 31)	0.13 (0.12 to 0.14)	0.13 (0.12 to 0.13)	.35	−0.01 (−0.02 to 0.01)
G_0 + T mixed (*n* = 65)	0.14 (0.13 to 0.14)	0.13 (0.12 to 0.13)	**<.001**	−0.01 (−0.02 to −0.01)
G_0 + T mixed + G (*n* = 18)	0.14 (0.14 to 0.15)	0.13 (0.12 to 0.14)	**.04**	−0.01 (−0.02 to −0.01)
Fiber density (× 10^22^ Da/cm^3^)^a^^,^^b^^,^^c^^,^^d^				
T total (*n* = 295)	4.4 (4.0 to 4.8)	4.7 (4.2 to 5.5)	**<.001**	0.3 (−0.1 to 1.0)
T i.m. undecanoate (*n* = 74)	4.5 (4.1 to 5.1)	5.0 (4.4 to 5.6)	**.002**	0.4 (−0.1 to 0.9)
T i.m. ester (*n* = 44)	4.4 (3.8 to 4.8)	4.5 (4.0 to 5.1)	**.01**	0.4 (−0.2 to 0.9)
T transdermal (*n* = 36)	4.2 (3.9 to 4.8)	5.0 (4.3 to 6.3)	**<.001**	0.8 (0.2 to 1.5)
T mixed (*n* = 27)	4.4 (4.1 to 4.8)	4.6 (4.1 to 5.1)	.10	0.2 (−0.4 to 0.9)
T mixed + G (*n* = 31)	4.2 (3.8 to 4.6)	4.6 (4.0 to 5.1)	.24	0.2 (−0.1 to 0.8)
G_0 + T mixed (*n* = 65)	4.4 (4.0 to 4.7)	4.6 (4.2 to 5.5)	**.005**	0.3 (−0.2 to 0.8)
G_0 + T mixed + G (*n* = 18)	4.2 (3.7 to 4.2)	4.4 (3.8 to 5.2)	.07	0.3 (−0.1 to 0.8)
Fibrinogen (g/L)^a^				
T total (*n* = 318)	3.0 (2.9 to 3.1)	2.9 (2.8 to 2.9)	**<.001**	−0.1 (−0.2 to −0.1)
T i.m. undecanoate (*n* = 83)	3.0 (2.9 to 3.1)	2.8 (2.7 to 2.9)	**.02**	−0.2 (−0.3 to −0.0)
T i.m. ester (*n* = 47)	3.1 (2.9 to 3.3)	2.9 (2.7 to 3.1)	.06	−0.2 (−0.3 to 0.0)
T transdermal (*n* = 39)	2.8 (2.6 to 3.1)	2.7 (2.6 to 2.9)	.20	−0.1 (−0.3 to 0.1)
T mixed (*n* = 32)	3.0 (2.8 to 3.1)	2.9 (2.8 to 3.1)	.93	−0.0 (−0.2 to 0.2)
T mixed + G (*n* = 33)	3.1 (2.9 to 3.4)	2.9 (2.7 to 3.1)	**.03**	−0.2 (−0.4 to −0.0)
G_0 + T mixed (*n* = 66)	3.0 (2.9 to 3.2)	2.8 (2.7 to 3.0)	**.008**	−0.2 (−0.3 to −0.1)
G_0 + T mixed + G (*n* = 18)	3.2 (2.8 to 3.6)	3.1 (2.8 to 3.5)	.48	−0.1 (−0.2 to 0.1)

Values presented as mean (95% CI) were compared at 0 and 12 months (within-group changes) with a paired *t*-test. Changes (0-12 months) are presented as mean (95% CI). Between-group comparisons (between gender-affirming hormone treatment administration forms) at 0 months were performed with an analysis of variance. Between-group comparisons at 12 months were performed with a linear regression analysis, adjusted for baseline values (0 months). Changes (0-12 months) were compared between gender-affirming hormone treatment administration forms using multivariate linear regression analysis. When significant between-group effects were observed in the analysis of variance or linear regression analysis, pairwise comparisons were performed with the Bonferroni test. *P* values shown in bold are statistically significant (*P* <.05).

G, injected, intrauterine, or oral progestin; G_0, progestin at baseline; i.m., intramuscular; MA, maximum absorbance; OD, optical density; OHP, overall hemostasis potential; T mixed, testosterone undecanoate or ester, or gel shifting over time; T, testosterone.

a Between-group comparisons at 12 months were performed with a multivariate linear regression analysis with log-transformed outcome variable or robust SEs to meet model assumptions.

b Changes (0-12 months) were compared between gender-affirming hormone treatment administration forms using multivariate linear regression analysis with robust SEs to meet model assumptions.

c Values presented as median (25, 75 percentiles) were compared at 0 and 12 months (within-group changes) with a Wilcoxon signed-rank test and between-group comparisons at 0 months were performed with a Kruskal–Wallis test.

d Changes (0-12 months) are presented as median (25, 75 percentiles).

We observed no between-group differences at 12 months, and there were no significant between-group differences in either absolute changes (Δ0-12 months; Table 3) or relative changes (Δ0-12 months/0 months; Figure). Normalization to fibrinogen resulted in longitudinal changes that were comparable with the unadjusted analysis, with consistent directions despite minor differences in statistical significance. No significant between-group differences were observed for absolute changes, also comparable with the unadjusted results. Fibrinogen-normalized results are shown in Supplementary Table 5.

## 4 Discussion

This is the first prospective study to investigate the effects of feminizing and masculinizing GAHT on *ex vivo* plasma fibrin clot characteristics. In transgender women, we demonstrated increased fibrin formation and reduced fibrin clot lysis, corresponding to a prothrombotic fibrin clot profile. These effects were most pronounced with oral administration. In transgender men, masculinizing GAHT was associated with reduced fibrin formation and an increased fibrin clot lysis, indicating an antithrombotic fibrin clot profile, with no differences between i.m. and transdermal treatment. These findings support our hypothesis about prothrombotic effects of GAHT in transgender women, whereas no prothrombotic effects were observed in transgender men.

In transgender women, increases in *V*_max_, MA, and OHP, together with reduced percentage clot lysis, indicate a faster and more extensive fibrin formation and reduced susceptibility to clot lysis following feminizing GAHT. Fiber diameter and fiber mass density remained unchanged, suggesting that feminizing GAHT primarily affected clot formation and lysis rather than the underlying fibrin structure. In comparison, Lim et al. [19] investigated the turbidimetric fibrin clot variable OHP in 26 transgender women receiving estradiol therapy (16 oral and 10 transdermal, with 14 also receiving CPA). They reported no significant difference in OHP compared with 55 cisgender men [19]. This discrepancy may relate to differences in study design. Our study also included a larger sample size and multiple clot variables. In contrast to our findings, Sidelmann et al. [20] found increased fiber diameter and fiber mass density in a longitudinal study of oral contraceptive use in healthy cisgender women. Differences in estrogen formulation and hormonal context may contribute to these divergent observations.

In transgender men, decreases in *V*_max_, MA, and OHP, together with increased percentage clot lysis, indicate slower and reduced fibrin formation and enhanced susceptibility to lysis following masculinizing GAHT. Fibrin fiber diameter decreased and fiber mass density increased, indicating structural changes with thinner, more compact fibers, tentatively pointing to tighter protofibril packing and less fiber hydration [29,30]. To our knowledge, no previous studies have examined fibrin clot characteristics in transgender men receiving testosterone. A case–control study in cisgender men demonstrated that fibrin clot lysis was lower in current abusers of anabolic androgenic steroids (including testosterone) than in former and never-users [21]. This discrepancy may relate to differences in study population and exposure, as individuals in the study by Sidelmann et al. [21] were older and used supraphysiological doses of mixed androgen formulations, resulting in markedly higher circulating estradiol levels than in former and never-users [21].

The present study applied a global plasma assay to assess fibrin formation and susceptibility to lysis. In the analytical setup, clot formation is initiated by excess thrombin and fibrinolysis by excess tissue plasminogen activator, thereby reducing the influence of the upstream coagulation cascade and interindividual variations in fibrinolytic activators or inhibitors. Consequently, the observed changes in clot characteristics are likely mediated primarily through alterations in the plasma concentration or composition of fibrinogen. Fibrinogen is synthesized in the liver [31], and since hepatocytes express estrogen and androgen receptors, GAHT may directly affect hepatic protein synthesis. Previous studies on estradiol therapy, including both oral contraceptive use in cisgender women and GAHT in transgender women, found increased fibrinogen levels during treatment [16,17,20,32]. We found the most pronounced effect after oral administration, likely due to first-pass hepatic metabolism and thus greater hormonal stimulation of hepatic protein synthesis [33]. As all transgender women in our study received combined estrogen and CPA, it is not possible to determine whether the observed prothrombotic effects are attributable to estrogen alone or whether CPA exerts independent or synergistic effects. Studies in cisgender men have reported decreased fibrinogen levels with various testosterone supplementation regimens [34], consistent with our findings in transgender men. However, a recent systematic review and meta-analysis in transgender men found no effect of 12 months of testosterone on fibrinogen levels [18].

When fibrin clot properties were normalized to fibrinogen levels, the overall pattern of changes persisted, indicating that the observed changes over time and between-group differences in clot characteristics cannot be explained solely by alterations in fibrinogen concentrations. This suggests that qualitative changes in fibrinogen composition may also contribute to the observed changes. This might reflect hormone-induced effects on hepatic synthesis of fibrinogen splice variants and/or posttranslational modifications known to affect fibrin clot properties [35].

In transgender women, the observed prothrombotic fibrin clot profile may represent a mechanistic link to the heightened thrombotic risk reported in this population. These results support previous results on thrombin generation in the same transgender cohort, showing that feminizing GAHT was procoagulant [8]. In contrast, GAHT-induced changes in fibrin clot properties and thrombin generation [8] suggest anticoagulant effects in transgender men and do not appear to explain the elevated thrombotic risk. Additional factors may therefore contribute. In our cohort, increases in body weight, total cholesterol, LDL cholesterol, triglycerides, and hematocrit were observed in transgender men (Supplementary Tables 2 and 3), factors that have been associated with thrombotic risk [36].

Our findings on fibrin clot characteristics and thrombin generation may contribute to improving clinical care and evidence-based guidelines for thromboprophylaxis in transgender individuals. Specifically, oral estradiol was associated with more pronounced prothrombotic changes compared with transdermal estradiol. This supports current clinical recommendations favoring transdermal administration in transgender women with elevated cardiovascular risk, such as individuals over 45 years of age or those with hyperlipidemia, hypertension, or a history of thromboembolic events [3]. In transgender men, no differences were observed between testosterone formulations.

An important strength of this study is the large cohort of transgender women and transgender men with paired blood samples obtained before and after 12 months of exposure to different GAHT regimens. Furthermore, multiple fibrin clot variables were assessed, providing a comprehensive evaluation of fibrin formation and lysis. This study also has limitations. The plasma-based assay does not account for potential effects mediated by blood cells. Furthermore, clot formation was assessed using thrombin activation, whereas the use of tissue factor, which activates the coagulation cascade at an earlier stage, might have revealed additional differences between groups. Plasma fibrin clot characteristics were not defined as primary outcome measures in the original study [23], and the present study should be regarded as a pilot study. The sample size may not have been sufficient to detect statistically significant differences within and between some of the smaller GAHT subgroups. In addition, fibrin network architecture was not directly assessed. Ideally, techniques such as scanning electron microscopy could provide complementary structural information, but this was not feasible due to limited plasma availability. Finally, baseline differences between GAHT groups were present for some clot characteristics, age, and cholesterol levels in transgender women. These baseline differences were adjusted for in the statistical analyses except for cholesterol, which was highly correlated with age (*r* = 0.5).

We conclude that in transgender women, both oral and transdermal estradiol combined with CPA were associated with a prothrombotic fibrin clot profile, which was least pronounced, and thus most favorable, following transdermal administration. In transgender men, each GAHT modality was associated with an antithrombotic fibrin clot profile, with no differences between i.m. and transdermal routes. By elucidating mechanisms linking GAHT to thrombotic risk, this large cohort study provides evidence that can optimize cardiovascular safety in transgender care.
