# Supplementary material for: Feminizing and masculinizing gender-affirming hormone therapy affects fibrin clot characteristics in opposite directions
Source: Res Pract Thromb Haemost. 2026 May 8;10(4):106648. doi: 10.1016/j.rpth.2026.106648 (PMC13240812; doi:10.1016/j.rpth.2026.106648)
Supplement: Supplementary Material [file mmc1.docx]

**Table S1. Comorbidities and medication use in transgender women and transgender men at baseline**

| COMORBIDITIES | Transgender women, n (%) | Transgender men, n (%) |
| --- | --- | --- |
| No comorbidity | 175 (70.0%) | 210 (65.6%) |
| Psychiatric and neurodevelopmental conditions   - Affective and anxiety-related disorders - Neurodevelopmental/neuropsychiatric disorders - Personality disorders | 62 (24.7%)  29 (11.5%)  30 (12.0%)  3 (1.2%) | 79 (24.7%)  45 (14.1%)  28 (8.8%)  6 (1.9%) |
| Allergic and atopic conditions   - Asthma - Allergic rhinitis | 10 (4.0%)  8 (3.2%)  2 (0.8%) | 29 (9.1%)  23 (7.2%)  6 (1.9%) |
| Dermatological conditions   - Acne - Eczema - Psoriasis | 11 (4.4%)  1 (0.4%)  5 (2.0%)  5 (2.0%) | 7 (2.2%)  0 (0.0%)  6 (1.9%)  1 (0.3%) |
| Neurological conditions   - Migraine - Epilepsy - Multiple sclerosis - Cerebral palsy | 8 (3.2%)  6 (2.4%)  1 (0.4%)  0 (0.0%)  1 (0.4%) | 14 (4.4%)  9 (2.8%)  2 (0.6%)  1 (0.3%)  2 (0.6%) |
| Pain and inflammatory conditions   - Rheumatoid arthritis - Fibromyalgia | 1 (0.4%)  1 (0.4%)  0 (0.0%) | 2 (0.6%)  1 (0.3%)  1 (0.3%) |
| Gastrointestinal conditions   - Inflammatory bowel disease - Irritable bowel syndrome | 6 (2.4%)  2 (0.8%)  4 (1.6%) | 9 (2.8%)  2 (0.6%)  7 (2.2%) |
| Endocrine and metabolic conditions   - Thyroid disease - Diabetes mellitus type 1 - Diabetes mellitus type 2 | 4 (1.6%)  3 (1.2%)  0 (0.0%)  2 (0.8%) | 5 (1.6%)  3 (0.9%)  1 (0.3%)  1 (0.3%) |
| Gynecological and hormonal conditions   - Polycystic ovary syndrome - Endometriosis | 0 (0.0%) | 7 (2.2%)  5 (1.6%)  2 (0.6%) |
| Cardiovascular conditions   - Hypertension - Familial hypercholesterolemia | 2 (0.8%)  1 (0.4%)  1 (0.4%) | 0 (0.0%) |
| Bone health conditions   - Osteoporosis | 1 (0.4%)  1 (0.4%) | 0 (0.0%) |
| Infectious diseases   - HIV infection | 1 (0.4%)  1 (0.4%) | 0 (0.0%) |
| MEDICATION USE |  |  |
| No medication other than GAHT | 172 (68.5%) | 215 (67.2%) |
| Psychotropic medication   - Antidepressants - Antipsychotics - ADHD medication - Anxiolytic / sedative / sleep-related | 40 (15.9%)  22 (8.8%)  1 (0.4%)  10 (4.0%)  7 (2.8%) | 81 (25.3%)  49 (15.3%)  12 (3.8%)  11 (3.4%)  9 (2.8%) |
| Allergy and airway medication   - Bronchodilators - Inhaled corticosteroids - Combination inhalers - Systemic antihistamines - Intranasal corticosteroids | 14 (5.6%)  6 (2.4%)  3 (1.2%)  1 (0.4%)  5 (2.0%)  2 (0.8%) | 30 (9.4%)  13 (4.1%)  7 (2.2%)  1 (0.3%)  15 (4.7%)  1 (0.3%) |
| Dermatological medication   - Topical corticosteroids - Topical acne treatment - Systemic acne treatment - Antifungal treatment - Systemic antibiotics | 13 (5.2%)  5 (2.0%)  1 (0.4%)  3 (1.2%)  2 (0.8%)  3 (1.2%) | 3 (0.9%)  2 (0.6%)  0 (0.0%)  0 (0.0%)  0 (0.0%)  1 (0.3%) |
| Neurological medication   - Antiepileptic medication - Antimigraine agents | 3 (1.2%)  1 (0.4%)  2 (0.8%) | 4 (1.3%)  3 (0.9%)  1 (0.3%) |
| Pain and anti-inflammatory medication   - Analgetics - NSAIDs - Opioids - Systemic corticosteroids - Disease-modifying anti-inflammatory drugs | 7 (2.8 %)  1 (0.4%)  3 (1.2%)  1 (0.4%)  2 (0.8%)  2 (0.8%) | 17 (5.3%)  5 (1.6%)  8 (2.5%)  2 (0.6%)  2 (0.6%)  1 (0.3%) |
| Gastrointestinal medication   - Proton pump inhibitors - Treatment for inflammatory bowel disease - Antispasmodics - Laxatives and fiber supplements | 12 (4.8%)  7 (2.8%)  1 (0.4%)  2 (0.8%)  2 (0.8%) | 13 (4.1%)  10 (3.1%)  2 (0.6%)  1 (0.3%)  5 (1.6%) |
| Endocrine and metabolic medication   - Thyroid hormone replacement - Antithyroid agents - Biguanides - Insulin | 5 (2.0%)  4 (1.6%)  1 (0.4%)  1 (0.4%)  1 (0.4%) | 4 (1.3%)  1 (0.3%)  1 (0.3%)  2 (0.6%)  0 (0.0%) |
| Cardiovascular medication   - Beta-blockers - Angiotensin II receptor blockers - Diuretics - Calcium canal blockers - Sympathomimetics | 3 (1.2%)  0 (0.0%)  1 (0.4%)  1 (0.4%)  1 (0.4%)  1 (0.4%) | 3 (0.9%)  3 (0.9%)  0 (0.0%)  0 (0.0%)  0 (0.0%)  0 (0.0%) |
| Bone health and dietary supplements   - Vitamin D - Calcium + vitamin D - Bisphosphonates - Iron supplements - Folic acid - Vitamin B12 - Multivitamin/mineral supplements | 13 (5.2%)  5 (2.0%)  1 (0.4%)  0 (0.0%)  0 (0.0%)  1 (0.4%)  1 (0.4%)  5 (2.0%) | 15 (4.7%)  6 (1.9%)  4 (1.3%)  1 (0.3%)  2 (0.6%)  0 (0.0%)  0 (0.0%)  1(0.3%) |
| Antiandrogen therapy*   - 5-α-reductase inhibitors | 9 (3.6%) | 0 (0.0%) |
| Anti-infective and immunological therapy   - Antiretroviral therapy - Immunoglobulin therapy | 2 (2.8%)  1 (0.4%)  1 (0.4%) | 0 (0.0%) |
| Urological and renal medication   - Antidiuretic hormone analogues | 1 (0.4%)  1 (0.4%) | 0 (0.0%) |

Affective and anxiety-related disorders include depression, bipolar disorder, anxiety disorders, obsessive-compulsive disorder, and posttraumatic stress disorder. Neurodevelopmental/neuropsychiatric disorders include attention-deficit/hyperactivity disorder (ADHD/ADD), autism spectrum disorder, and Tourette syndrome. Personality disorders include borderline personality disorders.
*Use of antiandrogen therapy other than cyproterone acetate during hormone treatment constituted an exclusion criterion. Nine participants received 5-α-reductase inhibitors at baseline prior to initiation of therapy.

**Table S2. Transgender women’s characteristics before and 12 months after gender affirming hormone therapy**

| Variable | 0 months | 12 months | P | Changes (0-12 months) |
| --- | --- | --- | --- | --- |
| **BMI** (kg/m^2^)^a^  - E total (n=268)  - E oral + CPA (n=173)  - E transdermal + CPA (n=77)  - E oral/transdermal + CPA (n=18) | 22.8 (22.4;23.2)  22.5 (22.0;22.9)  23.7 (22.9;24.6)^d^  21.8 (20.2;23.6) | 23.7 (23.3;24.1)  23.4 (22.9;23.9)  24.6 (23.8;25.4)  22.6 (21.0;24.3) | **<0.001**  **<0.001**  **<0.001**  **0.03** | 0.9 (0.7;1.2)  1.0 (0.6;1.3)  0.8 (0.4;1.2)  0.8 (0.1;1.5) |
| **Total testosterone** (nmol/l)^b,c^  - E total (n=256)  - E oral + CPA (n=166)  - E transdermal + CPA (n=74)  - E oral/transdermal + CPA (n=16) | 19.0 (14.8;23.0)  19.5 (15.0;23.1)  17.6 (14.0;23.3)  19.0 (14.3;23.1) | 0.6 (0.5;0.8)  0.6 (0.4;0.9)  0.6 (0.5;0.7)  0.5 (0.5;0.9) | **<0.001**  **<0.001**  **<0.001**  **<0.001** | -17.8 (-22.4;-13.8)  -18.9 (-22.4;-13.7)  -17.1 (-22.5;-13.7)  -18.1 (-22.6;-13.5) |
| **SHBG** (pmol/l)^a,c^  - E total (n=192)  - E oral + CPA (n=131)  - E transdermal (n=49)  - E oral/transdermal + CPA (n=12) | 34.5 (32.5;36.6)  34.6 (32.3;37.0)  34.0 (29.7;39.0)  35.7 (28.1;45.3) | 44.3 (41.2;47.7)  49.6 (45.8;53.8)  32.8 (28.4;37.9)^d^  44.0 (31.8;60.7) | **<0.001**  **<0.001**  0.50  0.13 | 7.9 (-2.0;23.6)  17.0 (4.0;27.5)  -2.0 (-8.1;4.6)^d^  5.7 (-2.7;20.5) |
| **17β-Estradiol** (pmol/l)^a,c^  - E total (n=256)  - E oral + CPA (n=166)  - E transdermal + CPA (n=74)  - E oral/transdermal + CPA (n=16) | 97 (94;101)  101 (97;107)  92 (85;99)  85 (73;99) | 221 (204;240)  205 (188;223)  252 (212;300)  260 (150;451) | **<0.001**  **<0.001**  **<0.001**  **<0.001** | 125 (50;238)  111 (43;202)  153 (71;340)^d^  101 (37;525) |
| **Cholesterol** (mmol/l)  - E total (n=254)  - E oral + CPA (n=165)  - E transdermal + CPA (n=74)  - E oral/transdermal + CPA (n=15) | 4.4 (4.3;4.6)  4.2 (4.1;4.4)  4.8 (4.6;5.1)^d^  4.5 (4.0;5.0) | 4.0 (3.9;4.1)  3.9 (3.8;4.0)  4.1 (3.9;4.3)^d^  3.9 (3.6;4.3) | **<0.001**  **<0.001**  **<0.001**  **0.008** | -0.5 (-0.5;-0.4)  -0.3 (-0.4;-0.3)  -0.7 (-0.9;-0.6)^d^  -0.6 (-1.0;-0.2) |
| **LDL cholesterol** (mmol/l)  - E total (n=254)  - E oral + CPA (n=165)  - E transdermal + CPA (n=74)  - E oral/transdermal + CPA (n=15) | 2.5 (2.4;2.7)  2.4 (2.3;2.5)  2.9 (2.6;3.1)^d^  2.6 (2.1;3.1) | 2.3 (2.2;2.4)  2.3 (2.1;2.4)  2.5 (2.3;2.7)  2.3 (2.0;2.7) | **<0.001**  **0.001**  **<0.001**  0.12 | -0.2 (-0.3;-0.2)  -0.1 (-0.2;-0.1)  -0.4 (-0.5;-0.3)^d^  -0.3 (-0.6;0.1) |
| **HDL cholesterol** (mmol/l)  - E total (n=254)  - E oral + CPA (n=165)  - E transdermal + CPA (n=74)  - E oral/transdermal + CPA (n=15) | 1.4 (1.3;1.4)  1.4 (1.3;1.4)  1.4 (1.3;1.5)  1.4 (1.2;1.6) | 1.2 (1.2;1.3)  1.2 (1.2;1.3)  1.2 (1.1;1.3)^d^  1.2 (1.1;1.4) | **<0.001**  **<0.001**  **<0.001**  **0.03** | -0.2 (-0.2;-0.1)  -0.1 (-0.2;-0.1)  -0.2 (-0.3;-0.2)^d^  -0.2 (-0.3;-0.0) |
| **Triglycerides** (mmol/l)^a,c^  - E total (n=254)  - E oral + CPA (n=165)  - E transdermal + CPA (n=74)  - E oral/transdermal + CPA (n=15) | 1.0 (0.9;1.0)  0.9 (0.9;1.0)  1.1 (0.9;1.2)  1.0 (0.8;1.4) | 0.8 (0.8;0.9)  0.8 (0.8;0.9)  0.9 (0.8;1.0)  0.8 (0.7;1.0) | **<0.001**  **<0.001**  **<0.001**  **0.07** | -0.1 (-0.4;0.1)  -0.1 (-0.4;0.1)  -0.2 (-0.5;0.0)  -0.3 (-0.4;-0.0) |
| **Hematocrit** (%)^c^  - E total (n=179)  - E oral + CPA (n=126)  - E transdermal + CPA (n=42)  - E oral/transdermal + CPA (n=11) | 45 (44;45)  45 (44;46)  44 (43;46)  44 (43;46) | 41 (41;41)  41 (40;41)  41 (40;42)  40 (39;41) | **<0.001**  **<0.001**  **0.001**  **<0.001** | -4 (-6;-2)  -4 (-5;-3)  -4 (-6;-1)  -5 (-6;-3) |

Participants’ characteristics were previously reported by Bøgehave et al. [8], van Velzen et al. [27] and Wierckx et al. [28].

Values presented as mean (95% CI) were compared at 0 and 12 months (within-group changes) with a paired t-test. Changes (0-12 months) are presented as mean (95% CI). Between-group comparisons (between GAHT administration forms) at baseline and for changes were performed with an ANOVA with post hoc Bonferroni tests for normally distributed variables and a Kruskal–Wallis test with post hoc Mann–Whitney tests for skewed variables. Between-group comparisons at 12 months were performed with an ANCOVA, adjusted for baseline values (0 months). When significant between-group effects were observed in the ANCOVA, pairwise comparisons were performed with the Bonferroni test.

ANCOVA, analysis of covariance; ANOVA, analysis of variance; BMI, body mass index; CPA, cyproterone acetate; E, estradiol; GAHT, gender-affirming hormone therapy; HDL, high-density lipoprotein; LDL, low-density lipoprotein; oral/transdermal, oral and transdermal estradiol shifting over time; SHBG, sex hormone binding globulin.

^a^ Values presented as geometric mean (95% CI) were compared at 0 and 12 months (within-group changes) with a paired t-test.

^b^ Values presented as median (25, 75 percentile) were compared at 0 and 12 months with a Wilcoxon test.

^c^ Changes (0-12 months) are presented as median (25, 75 percentiles).

^d^ Significantly different from E oral + CPA.

**Table S3. Transgender men’s characteristics before and 12 months after gender affirming hormone therapy**

| Variable | 0 months | 12 months | P | Changes (0-12 months) |
| --- | --- | --- | --- | --- |
| **BMI** (kg/m^2^)^a^  - T total (n=342)  - T IM undecanoate (n=85)  - T IM ester (n=49)  - T transdermal (n=43)  - T mixed (n=39) | 24.0 (23.6;24.5)  23.4 (22.6;24.1)  24.7 (23.5;26.1)  24.2 (22.8;25.6)  24.8 (23.4;26.3) | 24.7 (24.3;25.1)  24.0 (23.3;24.7)  25.2 (24.0;26.4)  24.9 (23.6;26.2)  25.5 (24.2;26.9) | **<0.001**  **<0.001**  0.15  **0.004**  **0.02** | 0.6 (0.4;0.8)  0.6 (0.3;0.9)  0.4 (-0.3;1.0)  0.7 (0.2;1.2)  0.6 (0.0;1.3) |
| - T mixed + G (n=36) | 24.2 (22.9;25.6) | 25.2 (23.8;26.6) | **0.002** | 1.0 (0.3;1.6) |
| - G_0 + T mixed (n=68) | 23.5 (22.6;24.5) | 24.2 (23.3;25.1) | **0.007** | 0.6 (0.1;1.1) |
| - G_0 + T mixed + G (n=22) | 24.8 (23.1;26.7) | 25.2 (23.6;27.0) | 0.41 | 0.4 (-0.6;1.3) |
| **Total testosterone** (nmol/l)^b,c^  - T total (n=323)  - T IM undecanoate (n=75)  - T IM ester (n=45)  - T transdermal (n=45)  - T mixed (n=38)  - T mixed + G (n=36)  - G_0 + T mixed (n=62)  - G_0 + T mixed + G (n=22) | 1.2 (0.9;1.5)  1.1 (0.8;1.5)  1.2 (1.0;1.6)  1.1 (0.9;1.4)  1.2 (1.0;1.5)  1.4 (0.9;1.8)^d^  1.1 (0.7;1.4)  1.4 (1.1;1.9)^e^ | 22.0 (15.0;30.2)  22.6 (16.2;27.3)  26.0 (17.0;36.5)  23.0 (14.0;37.0)  19.5 (14.8;32.5)  19.6 (13.4;25.3)  20.2 (15.0;27.3)  25.8 (15.7;51.3) | **<0.001**  **<0.001**  **<0.001**  **<0.001**  **<0.001**  **<0.001**  **<0.001**  **<0.001** | 20.7 (13.9;29.0)  21.1 (15.0;26.2)  25.4 (15.2;35.3)  22.1 (13.0;36.1)  18.4 (13.4;30.7)  18.7 (11.6;24.3)  18.9 (14.2;26.7)  24.6 (14.4;50.0) |
| **SHBG** (pmol/l)^a,c^  - T total (n=259)  - T IM undecanoate (n=70)  - T IM ester (n=34)  - T transdermal (n=38)  - T mixed (n=30)  - T mixed + G (n=23)  - G_0 + T mixed (n=52)  - G_0 + T mixed + G (n=12) | 46.3 (42.8;50.1)  53.9 (46.0;63.1)  42.5 (36.9;49.0)  50.7 (43.0;59.7)  44.6 (37.2;53.2)  62.7 (49.6;79.3)  39.0 (31.1;48.8)  24.0 (15.4;37.5)^f^ | 27.2 (25.7;28.7)  29.1 (26.4;32.1)  21.6 (18.2;25.6)  26.5 (23.4;30.0)  23.2 (19.9;26.9)  31.2 (25.9;37.6)  30.5 (26.7;34.9)^h^  26.3 (18.2;38.0) | **<0.001**  **<0.001**  **<0.001**  **<0.001**  **<0.001**  **<0.001**  0.06  0.71 | -20.0 (-35.0;-7.0)  -23.0 (-55.9;-6.9)  -21.5 (-29.3;-11.8)  -28.0 (-32.5;-15.5)  -19.5 (-35.0;-12.0)  -29.7 (-55.0;-14.9)  -10.3 (-38.7;13.7)^g^  3.8 (-5.8;11.0)^g^ |
| **17β-Estradiol** (pmol/l)^a,c^  - T total (n=321)  - T IM undecanoate (n=74)  - T IM ester (n=45)  - T transdermal (n=45)  - T mixed (n=38)  - T mixed + G (n=36)  - G_0 + T mixed (n=62)  - G_0 + T mixed + G (n=21) | 195 (175;218)  215 (179;258)  226 (170;301)  242 (184;318)  301 (206;439)  305 (244;393)  114 (91;144)^g^  68 (45;104)^g^ | 155 (147;164)  150 (133;169)  157 (137;180)  164 (142;189)  150 (128;175)  170 (139;209)  147 (133;163)  157 (122;203) | **<0.001**  **<0.001**  **0.02**  **0.01**  **<0.001**  **0.001**  0.05  **<0.001** | -28 (-258;59)  -39 (-264;31)  -60 (-243;45)  -70 (-355;49)  -172 (-643;-4)  -214 (-439;7)  17 (-47;90)^g^  101 (24;154)^g^ |
| **Cholesterol** (mmol/l)  - T total (n=334)  - T IM undecanoate (n=80)  - T IM ester (n=47)  - T transdermal (n=45)  - T mixed (n=39)  - T mixed + G (n=34)  - G_0 + T mixed (n=67)  - G_0 + T mixed + G (n=22) | 4.4 (4.3;4.4)  4.6 (4.4;4.7)  4.3 (4.1;4.6)  4.4 (4.1;4.6)  4.2 (4.0;4.4)  4.2 (3.9;4.5)  4.3 (4.1;4.5)  4.4 (3.9;4.8) | 4.4 (4.4;4.5)  4.6 (4.4;4.8)  4.3 (4.1;4.6)  4.7 (4.4;4.9)  4.3 (4.0;4.6)  4.3 (4.0;4.7)  4.4 (4.1;4.6)  4.4 (4.0;4.8) | **0.006**  0.57  0.67  **<0.001**  0.31  0.11  0.14  0.82 | 0.1 (0.0;0.2)  0.1 (-0.1;0.2)  -0.0 (-0.2;0.1)  0.3 (0.1;0.4)  0.1 (-0.1;0.3)  0.1 (0.0;0.3)  0.1 (0.0;0.3)  0.0 (-0.3;0.3) |
| **LDL cholesterol** (mmol/l)  - T total (n=332)  - T IM undecanoate (n=78)  - T IM ester (n=47)  - T transdermal (n=45)  - T mixed (n=39)  - T mixed + G (n=34)  - G_0 + T mixed (n=67)  - G_0 + T mixed + G (n=22) | 2.4 (2.3;2.5)  2.5 (2.3;2.6)  2.4 (2.2;2.7)  2.3 (2.1;2.6)  2.3 (2.1;2.5)  2.2 (2.0;2.4)  2.5 (2.3;2.7)  2.8 (2.4;3.2) | 2.6 (2.6;2.7)  2.8 (2.6;3.0)  2.5 (2.3;2.8)  2.7 (2.5;2.9)  2.6 (2.3;2.8)  2.6 (2.3;2.9)  2.6 (2.4;2.8)  2.6 (2.2;3.0)^f^ | **<0.001**  **<0.001**  0.24  **<0.001**  **0.001**  **<0.001**  0.38  0.21 | 0.2 (0.2;0.3)  0.3 (0.2;0.4)  0.1 (-0.1;0.3)  0.4 (0.2;0.5)  0.3 (0.1;0.5)  0.4 (0.3;0.6)  0.1 (-0.1;0.2)  -0.2 (-0.6;0.1)^f^ |
| **HDL cholesterol** (mmol/l)  - T total (n=333)  - T IM undecanoate (n=79)  - T IM ester (n=47)  - T transdermal (n=45)  - T mixed (n=39)  - T mixed + G (n=34)  - G_0 + T mixed (n=67)  - G_0 + T mixed + G (n=22) | 1.5 (1.5;1.6)  1.6 (1.6;1.7)  1.4 (1.3;1.6)  1.6 (1.5;1.7)  1.5 (1.4;1.7)  1.7 (1.5;1.8)  1.4 (1.3;1.5)^f^  1.2 (1.1;1.3)^f^ | 1.3 (1.3;1.3)  1.3 (1.2;1.4)  1.2 (1.1;1.2)  1.2 (1.2;1.5)  1.2 (1.1;1.3)  1.3 (1.2;1.5)  1.3 (1.2;1.4)^i^  1.3 (1.2;1.5)^i^ | **<0.001**  **<0.001**  **<0.001**  **<0.001**  **<0.001**  **<0.001**  0.09  0.22 | -0.2 (-0.3;-0.2)  -0.3 (-0.4;-0.3)  -0.3 (-0.4;-0.2)  -0.2 (-0.3;-0.1)  -0.3 (-0.4;-0.2)  -0.3 (-0.4;-0.2)  -0.1 (-0.1;0.0)^i^  0.1 (-0.1;0.3)^i^ |
| **Triglycerides** (mmol/l)^a,c^  - T total (n=333)  - T IM undecanoate (n=79)  - T IM ester (n=47)  - T transdermal (n=45)  - T mixed (n=39)  - T mixed + G (n=34)  - G_0 + T mixed (n=67)  - G_0 + T mixed + G (n=22) | 0.8 (0.7;0.8)  0.8 (0.7;0.9)  0.9 (0.8;1.1)  0.8 (0.7;0.9)  0.8 (0.8;0.9)  0.7 (0.7;0.8)  0.7 (0.6;0.8)  0.7 (0.6;0.9) | 1.0 (1.0;1.0)  1.0 (0.9;1.1)  1.1 (1.0;1.3)  1.1 (1.0;1.3)  1.0 (0.9;1.1)  0.8 (0.7;1.0)  0.9 (0.8;1.0)  1.0 (0.8;1.2) | **<0.001**  **<0.001**  **<0.001**  **<0.001**  **<0.003**  0.11  **<0.001**  **<0.001** | 0.2 (0.0;0.4)  0.2 (-0.1;0.4)  0.2 (-0.1;0.6)  0.3 (0.0;0.6)  0.2 (-0.1;0.4)  0.1 (-0.1;0.3)  0.2 (0.0;0.4)  0.3 (0.0;0.4) |
| **Hematocrit** (%)  - T total (n=300)  - T IM undecanoate (n=75)  - T IM ester (n=38)  - T transdermal (n=40)  - T mixed (n=35)  - T mixed + G (n=31)  - G_0 + T mixed (n=63)  - G_0 + T mixed + G (n=18) | 40 (40;41)  40 (39;41)  40 (39;41)  39 (39;40)  41 (40;41)  40 (39;41)  41 (41;42)  43 (41;44) | 45 (45;46)  45 (45;46)  46 (45;47)  45 (44;46)  46 (45;47)  46 (44;47)  45 (44;46)  46 (45;47) | **<0.001**  **<0.001**  **<0.001**  **<0.001**  **<0.001**  **<0.001**  **<0.001**  **<0.001** | 5 (5;5)  5 (4;6)  6 (5;7)  5 (4;6)  5 (4;6)  6 (5;7)  4 (3;4)  3 (2;5) |

Participants’ characteristics were previously reported by Bøgehave et al. [8], van Velzen et al. [27] and Wierckx et al. [28].

Values presented as mean (95% CI) were compared at 0 and 12 months (within-group changes) with a paired t-test. Changes (0-12 months) are presented as mean (95% CI). Between-group comparisons (between GAHT administration forms) at baseline and for changes were performed with an ANOVA with post hoc Bonferroni tests for normally distributed variables and a Kruskal–Wallis test with post hoc Mann–Whitney tests for skewed variables. Between-group comparisons at 12 months were performed with an ANCOVA, adjusted for baseline values (0 months). When significant between-group effects were observed in the ANCOVA, pairwise comparisons were performed with the Bonferroni test.

ANCOVA, analysis of covariance; ANOVA, analysis of variance; BMI, body mass index; G, injected, intrauterine, or oral gestagen; G_0, gestagen at baseline; GAHT, gender-affirming hormone therapy; HDL, high-density lipoprotein; IM, intramuscular; LDL, low-density lipoprotein; SHBG, sex hormone binding globulin; T mixed, testosterone undecanoate or ester or gel shifting over time; T, testosterone.

^a^ Values presented as geometric mean (95% CI) were compared at 0 and 12 months (within-group changes) with a paired t-test.

^b^ Values presented as median (25, 75 percentiles) were compared at 0 and 12 months with a Wilcoxon test.

^c^ Changes (0-12 months) are presented as median (25, 75 percentiles).

^d^ Significantly different from T IM undecanoate, T transdermal, and G_0 + T mixed.

^e^ Significantly different from T transdermal and G_0 + T mixed.

^f^ Significantly different from T IM undecanoate, T transdermal, and T mixed + G.

^g^ Significantly different from T IM undecanoate, T IM ester, T transdermal, T mixed, and T mixed + G.

^h^ Significantly different from T IM ester and T mixed.

^i^ Significantly different from T IM undecanoate, T IM ester, T mixed, and T mixed + G.

**Table S4. Fibrin clot characteristics normalized to fibrinogen levels in transgender women before and 12 months after gender-affirming hormone therapy**

| Variable | 0 months | 12 months | P | Change (0-12 months) |
| --- | --- | --- | --- | --- |
| V_max_ (OD/min/[g/L])^a,b^ |  |  |  |  |
| - E total (n=249) | 0.26 (0.25;0.26) | 0.27 (0.27;0.28) | **<0.001** | 0.02 (0.01;0.02) |
| - E oral + CPA (n=163) | 0.26 (0.25;0.27) | 0.28 (0.27;0.29) | **<0.001** | 0.02 (0.01;0.02) |
| - E transdermal + CPA (n=69) | 0.25 (0.24;0.27) | 0.28 (0.26;0.29) | **<0.001** | 0.02 (0.01;0.03) |
| - E oral/transdermal + CPA (n=17) | 0.24 (0.21;0.27) | 0.25 (0.22;0.28) | 0.29 | 0.01 (-0.01;0.03) |
| MA (OD/[g/L]) |  |  |  |  |
| - E total (n=248) | 0.21 (0.21;0.21) | 0.22 (0.22;0.22) | **<0.001** | 0.009 (0.006;0.012 |
| - E oral + CPA (n=163) | 0.21 (0.21;0.22) | 0.22 (0.22;0.22) | **<0.001** | 0.007 (0.003;0.010) |
| - E transdermal + CPA (n=69) | 0.21 (0.21;0.22) | 0.23 (0.22;0.23) | **<0.001** | 0.014 (0.010;0.019)^d^ |
| - E oral/transdermal + CPA (n=16) | 0.20 (0.18;0.22) | 0.21 (0.20;0.22) | 0.13 | 0.011 (-0.002;0.024) |
| OHP (OD x min/[g/L]) |  |  |  |  |
| - E total (n=249) | 18.2 (17.8;18.6) | 19.5 (19.2;19.9) | **<0.001** | 1.3 (1.1;1.6) |
| - E oral + CPA (n=163) | 18.1 (17.6;18.5) | 19.4 (19.0;19.8) | **<0.001** | 1.4 (1.0;1.7) |
| - E transdermal + CPA (n=69) | 18.8 (18.0;19.5) | 20.1 (19.4;20.7) | **<0.001** | 1.3 (0.8;18) |
| - E oral/transdermal + CPA (n=17) | 17.2 (15.5;18.8) | 18.6 (17.3;19.9) | **0.04** | 1.4 (0.2;2.7) |
| Clot lysis (%/[g/L]) |  |  |  |  |
| - E total (n=249) | 30.5 (29.1;31.8) | 26.4 (25.3;27.5) | **<0.001** | -4.1 (-5.1;-3.0) |
| - E oral + CPA (n=163) | 32.7 (30.9;34.4) | 27.0 (25.6;28.5) | **<0.001** | -5.6 (-7.0;-4.3) |
| - E transdermal + CPA (n=69) | 25.7 (23.4;27.9)^c^ | 25.1 (23.1;27.1) | 0.46 | -0.6 (-2.1;0.96)^c^ |
| - E oral/transdermal + CPA (n=17) | 28.8 (24.4;33.2) | 25.6 (21.3;29.9) | **0.03** | -3.2 (-5.9;-0.5) |

Values presented as mean (95% CI) were compared at 0 and 12 months (within-group changes) with a paired t-test. Changes (0-12 months) are presented as mean (95% CI). Between-group comparisons (between GAHT administration forms) at 0 months were performed with an ANOVA. Between-group comparisons at 12 months were performed with a linear regression analysis, adjusted for baseline values (0 months) and age. Changes (0-12 months) were compared between GAHT administration forms using multivariate linear regression analysis. When significant between-group effects were observed in the ANOVA or linear regression analysis, pairwise comparisons were performed with the Bonferroni test.

ANOVA, analysis of variance; CPA, cyproterone acetate; E, estradiol; MA, maximum absorbance; OD, optical density; OHP, overall hemostasis potential; oral/transdermal, oral and transdermal estradiol shifting over time.

^a^Between-group comparisons at 12 months were performed with a multivariate linear regression analysis with log-transformed outcome variables or robust standard errors to meet model assumptions.

^b^Changes (0-12 months) were compared between GAHT administration forms using multivariate linear regression analysis with robust standard errors to meet model assumptions.

^c^Significantly lower than E oral + CPA.

^d^Significantly higher than E oral + CPA.

**Table S5. Fibrin clot characteristics normalized to fibrinogen levels in transgender men before and 12 months after gender-affirming hormone therapy**

| Variable | 0 months | 12 months | P | Change (0-12 months) |
| --- | --- | --- | --- | --- |
| V _max_ (OD/min/[g/L]) |  |  |  |  |
| - T total (n=318) | 0.26 (0.25;0.27) | 0.25 (0.25;0.26) | **0.003** | -0.007 (-0.01;-0.002) |
| - T IM undecanoate (n=83) | 0.25 (0.24;0.26) | 0.25 (0.24;0.26) | 0.75 | -0.002 (-0.011;0.008) |
| - T IM ester (n=47) | 0.26 (0.25;0.28) | 0.27 (0.25;0.28) | 0.69 | 0.002 (-0.008;0.012 |
| - T transdermal (n=39) | 0.28 (0.26;0.30) | 0.26 (0.25;0.28) | 0.09 | -0.012 (-0.025;0.001) |
| - T mixed (n=32) | 0.26 (0.24;0.28) | 0.25 (0.23;0.27) | 0.10 | -0.012 (-0.025;0.002) |
| - T mixed + G (n=33) | 0.25 (0.23;0.27) | 0.24 (0.22;0.27) | 0.13 | -0.009 (-0.021;0.002) |
| - G_0 + T mixed (n=66) | 0.26 (0.25;0.27) | 0.25 (0.23;0.26) | **0.026** | -0.011 (-0.020;-0.002) |
| - G_0 + T mixed + G (n=18) | 0.27 (0.25;0.29) | 0.26 (0.23;0.28) | 0.22 | -0.012 (-0.032;0.007) |
| MA (OD/[g/L]) |  |  |  |  |
| - T total (n=318) | 0.22 (0.22;0.22) | 0.21 (0.21;0.21) | **<0.001** | -0.011 (-0.014;-0.009) |
| - T IM undecanoate (n=83) | 0.21 (0.21;0.22)^a^ | 0.21 (0.20;0.21) | **0.001** | -0.008 (-0.012;-0.003) |
| - T IM ester (n=47) | 0.22 (0.22;0.23) | 0.21 (0.20;0.22) | **<0.001** | -0.011 (-0.017;-0.005) |
| - T transdermal (n=39) | 0.22 (0.21;0.23) | 0.20 (0.20;0.21) | **<0.001** | -0.014 (-0.020;-0.007) |
| - T mixed (n=32) | 0.22 (0.21;0.23) | 0.21 (0.20;0.22) | **0.01** | -0.011 (-0.020;-0.003) |
| - T mixed + G (n=33) | 0.22 (0.22;0.23) | 0.21 (0.20;0.22) | **<0.001** | -0.015 (-0.023;-0.007) |
| - G_0 + T mixed (n=66) | 0.22 (0.22;0.23) | 0.21 (0.20;0.21) | **<0.001** | -0.012 (-0.017;-0.008) |
| - G_0 + T mixed + G (n=18) | 0.23 (0.22;0.24) | 0.22 (0.21;0.23) | **0.01** | -0.016 (-0.027;-0.005) |
| OHP (OD x min/[g/L]) |  |  |  |  |
| - T total (n=318) | 20.0 (19.7;20.3) | 18.4 (18.1;18.7) | **<0.001** | -1.6 (-1.8;-1.3) |
| - T IM undecanoate (n=83) | 19.3 (18.8;19.8) | 18.0 (17.5;18.5) | **<0.001** | -1.3 (-1.8;-0.8) |
| - T IM ester (n=47) | 20.5 (19.7;21.3) | 18.7 (17.9;21.3) | **<0.001** | -1.8 (-2.4;-1.3) |
| - T transdermal (n=39) | 19.2 (18.2;20.2) | 17.9 (17.1;18.7) | **0.001** | -1.3 (-2.0;-0.6) |
| - T mixed (n=32) | 20.0 (19.0;21.1) | 18.6 (17.7;19.5) | **0.005** | -1.4 (-2.3;-0.5) |
| - T mixed + G (n=33) | 20.7 (19.8;21.7) | 18.8 (17.7;19.8) | **<0.001** | -2.0 (-2.9;-1.1) |
| - G_0 + T mixed (n=66) | 20.1 (19.5;20.8) | 18.4 (17.8;19.1) | **<0.001** | -1.7 (-2.2;-1.2) |
| - G_0 + T mixed + G (n=18) | 21.3 (19.9;22.7) | 19.2 (18.1;20.4) | **0.003** | -2.0 (-3.2;-0.9) |
| Clot lysis (%/[g/L]) |  |  |  |  |
| - T total (n=318) | 24.0 (23.0;25.0) | 27.1 (26.0;28.1) | **<0.001** | 3.1 (2.3;3.9) |
| - T IM undecanoate (n=83) | 24.3 (22.5;26.1) | 27.9 (26.0;29.7) | **<0.001** | 3.6 (1.7;5.4) |
| - T IM ester (n=47) | 22.4 (19.5;25.3) | 26.3 (23.5;29.1) | **<0.001** | 3.9 (1.8;6.0) |
| - T transdermal (n=39) | 27.9 (24.2;31.6) | 29.6 (26.3;32.9) | 0.21 | 1.7 (-0.9;4.2) |
| - T mixed (n=32) | 24.3 (21.6;27.0) | 25.2 (22.6;27.7) | 0.51 | 0.9 (-1.7;3.5) |
| - T mixed + G (n=33) | 22.0 (18.9;25.0) | 25.4 (22.5;28.2) | **<0.001** | 3.4 (1.7;5.2) |
| - G_0 + T mixed (n=66) | 23.6 (21.4;26.3) | 27.8 (25.1;30.5) | **<0.001** | 4.0 (2.2;5.7) |
| - G_0 + T mixed + G (n=18) | 21.8 (18.2;25.5) | 23.8 (20.1;27.5) | 0.08 | 2.0 (-0.1;4.0) |

Values presented as mean (95% CI) were compared at 0 and 12 months (within-group changes) with a paired t-test. Changes (0-12 months) are presented as mean (95% CI). Between-group comparisons (between GAHT administration forms) at 0 months were performed with an ANOVA. Between-group comparisons at 12 months were performed with a linear regression analysis, adjusted for baseline values (0 months). Changes (0-12 months) were compared between GAHT administration forms using multivariate linear regression analysis. When significant between-group effects were observed in the ANOVA or linear regression analysis, pairwise comparisons were performed with the Bonferroni test.

ANOVA, analysis of variance; G, injected, intrauterine, or oral progestin; G_0, progestin at baseline; IM, intramuscular; MA, maximum absorbance; OD, optical density; OHP, overall hemostasis potential; T mixed, testosterone undecanoate or ester or gel shifting over time; T, testosterone.

^a^Significantly lower than G_0 + T mixed + G


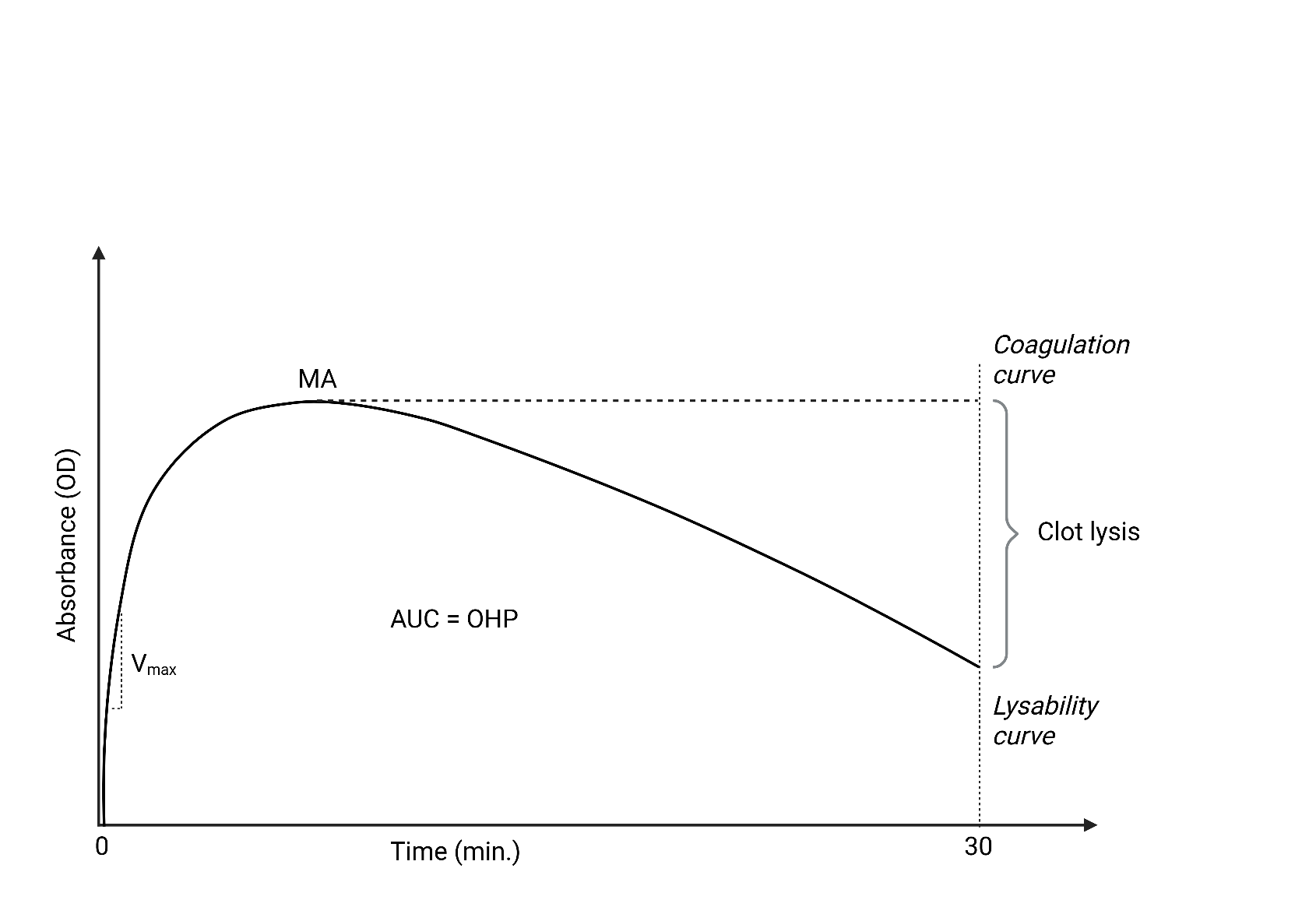


**Supplementary Figure**: Simplified illustration of the turbidity curve and the derived variables. The solid line represents the lysability curve, and the dotted line (long dashes) represents the coagulation curve. AUC, area under the curve; MA, maximum absorbance; OHP, overall hemostasis potential. Created in BioRender. Dippel, S. (2025) https://BioRender.com/1agxz2l.
